# COX-2 overexpression in resected pancreatic head adenocarcinomas correlates with favourable prognosis

**DOI:** 10.1186/1471-2407-14-458

**Published:** 2014-06-20

**Authors:** Ewa Pomianowska, Aasa R Schjølberg, Ole Petter F Clausen, Ivar P Gladhaug

**Affiliations:** 1 Institute of Clinical Medicine, Faculty of Medicine, University of Oslo, Oslo, Norway; 2 Department of Hepato-pancreato-biliary Surgery, Oslo University Hospital, Rikshospitalet, PO Box 4950 Nydalen, 0424 Oslo, Norway; 3 Department of Pathology, Oslo University Hospital, Rikshospitalet, Oslo, Norway

## Abstract

**Background:**

Overexpression of cyclooxygenase-2 (COX-2) has been implicated in oncogenesis and progression of adenocarcinomas of the pancreatic head. The data on the prognostic importance of COX expression in these tumours is inconsistent and conflicting. We evaluated how COX-2 overexpression affected overall postoperative survival in pancreatic head adenocarcinomas.

**Methods:**

The study included 230 consecutive pancreatoduodenectomies for pancreatic cancer (PC, n = 92), ampullary cancer (AC, n = 62) and distal bile duct cancer (DBC, n = 76). COX-2 expression was assessed by immunohistochemistry. Associations between COX-2 expression and histopathologic variables including degree of differentiation, histopathologic type of differentiation (pancreatobiliary vs. intestinal) and lymph node ratio (LNR) were evaluated. Unadjusted and adjusted survival analysis was performed.

**Results:**

COX-2 staining was positive in 71% of PC, 77% in AC and 72% in DBC. Irrespective of tumour origin, overall patient survival was more favourable in patients with COX-2 positive tumours than COX-2 negative (p = 0.043 in PC, p = 0.011 in AC, p = 0.06 in DBC). In tumours of pancreatobiliary type of histopathological differentiation, COX-2 expression did not significantly affect overall patient survival. In AC with intestinal differentiation COX-2 expression significantly predicted favourable survival (p = 0.003). In PC, COX-2 expression was significantly associated with high degree of differentiation (p = 0.002). COX-2 and LNR independently predicted good prognosis in a multivariate model.

**Conclusions:**

COX-2 is overexpressed in pancreatic cancer, ampullary cancer and distal bile duct cancer and confers a survival benefit in all three cancer types. In pancreatic cancer, COX-2 overexpression is significantly associated with the degree of differentiation and independently predicts a favourable prognosis.

## Background

Primary adenocarcinomas located in the pancreatic head arise from the ampulla, the distal bile duct, or the pancreatic ductal structures. Due to the topological proximity of these structures, resectable adenocarcinomas arising from any of these three anatomical locations are typically resected by the same surgical procedure, i.e. curative-intent pancreatoduodenectomy. The considerable variation in reported frequencies for the individual tumour sites suggests that the precise tumour origin may be difficult to determine [1] and that the applied methods for histopathological determination of the cancer origin varies widely among institutions [2]. Adenocarcinomas from all three locations may be of pancreatobiliary or intestinal type of differentiation [3].

Overexpression of cyclooxygenase-2 (COX-2) has been described in several tumours, including colon, stomach, breast, lung, and urinary bladder [4-16]. The COX-2 expression is a component of the cellular response to inflammation and is induced by several extracellular or intracellular stimuli, including proinflammatory cytokines, infectious agents, mitogens, hormones and growth factors [17,18]. Several studies have reported overexpression of COX-2 in subsets of pancreatic adenocarcinomas in 37 – 80% of the tumours investigated [19-26]. Increased COX-2 expression has also been demonstrated in pancreatic intraepithelial neoplasias (PanINs) [27-30]. However there is relatively few data on COX-2 expression in the two other types of pancreatic head adenocarcinomas, ampullary cancer [31-33] and distal bile duct cancer [34]. Data on prognostic relevance of COX-2 overexpression in all these tumours has been inconsistent and conflicting although most reports indicate an inverse relationship between COX-2 overexpression and survival rates in pancreatic cancer [19,21] and ampullary cancer [32].

The aim of the present study was to examine the prognostic relevance of COX-2 expression in adenocarcinomas from the three separate anatomical sites of origin in the pancreatic head. The data shows that COX-2 is overexpressed in all three types of pancreatic head adenocarcinomas and that COX-2 overexpression is associated with better survival. In contrast to previous reports, COX-2 overexpression was found to be an independent prognostic factor for better survival in pancreatic adenocarcinoma.

## Methods

### Patients

The study included 230 consecutive patients (103 women and 127 men) undergoing a standard Whipple’s procedure for adenocarcinoma with curative intent 1998 -2011 at Oslo University Hospital, Rikshospitalet. The study was approved by the Regional Committee for Medical and Health Research Ethical for Southern Norway.

Standard demographic, clinicopathological, and tumour-specific data were collected retrospectively from hospital records. Overall survival data was obtained from the Norwegian Population Registry, updated June 20, 2013. Since all Norwegian inhabitants receive a unique personal identification number, no patients were lost to follow-up in the present study. Patients were followed until death or censored after maximum five years (60 months). By the end of the study 177 patients were dead. Median follow-up for the remaining 53 patients was 62 months (interquartile range 29 -119 months). Perioperative death (defined as death within 30 days of operation) was included in the analyses (four patients). Analysis excluding perioperative death gave similar results. None of the patients received preoperative chemotherapy or chemoradiotherapy. From 2008, adjuvant chemotherapy with 5-fluororuracil was recommended for eligible patients operated for pancreatic cancer. Thirty-nine percent of the patients (13 of 33) operated in this period received adjuvant chemotherapy (5-FU-based in 11 patients, 2 patients received gemcitabine).

### Histopathological evaluation of resection specimens

The resection specimens were examined according to a standardized protocol as described previously [1,35]. All registered parameters of the prospectively collected data base, including anatomic site of tumour origin, where later reevaluated by slide review [1]. The histological type of differentiation was evaluated and all tumours were classified either as intestinal or pancreatobiliary type [3,36]. In brief, pancreatobiliary tumours typically have simple or branching glands and small solid nests of cells surrounded by a desmoplastic stroma, and have cuboideal to low columnar epithelium arranged in a single layer and the nuclei are rounded but with marked variation in size and shape from one cell to the next. Intestinal tumours typically resembled colon cancer, have tall and often pseudostratified columnar epithelium with oval nuclei located in the more basal aspect of the cytoplasm, and there may also often be presence of mucin [36,37].

### Immunohistochemistry

Formalin-fixed, paraffin-embedded tissue was sectioned (3 μm), dried at 60°C, and processed in a Ventana BenchMark Ultra machine (Ventana Medical Systems Inc. (Tucson Arizona USA). Slides were incubated with monoclonal anti-COX-2 antibodies (Thermo Fischer Scientific rabbit), Universal Alkaline Phosphatase Red Detection Kit (Ultra View 760-501) and αSMA (Dako M.0851), DAB (Ultra View 760-500). Additional immunostaining on duplicates of twenty slides was performed with monoclonal COX-2 mouse antibody Invitrogen (Camarillo, CA, USA). Slides were counterstained with haematoxylin, fixed, mounted and analyzed using an inverted light microscope (Olympus, Center Valley, PA, USA).

### Evaluation of COX-2 immunostaining

**Figure 1:**
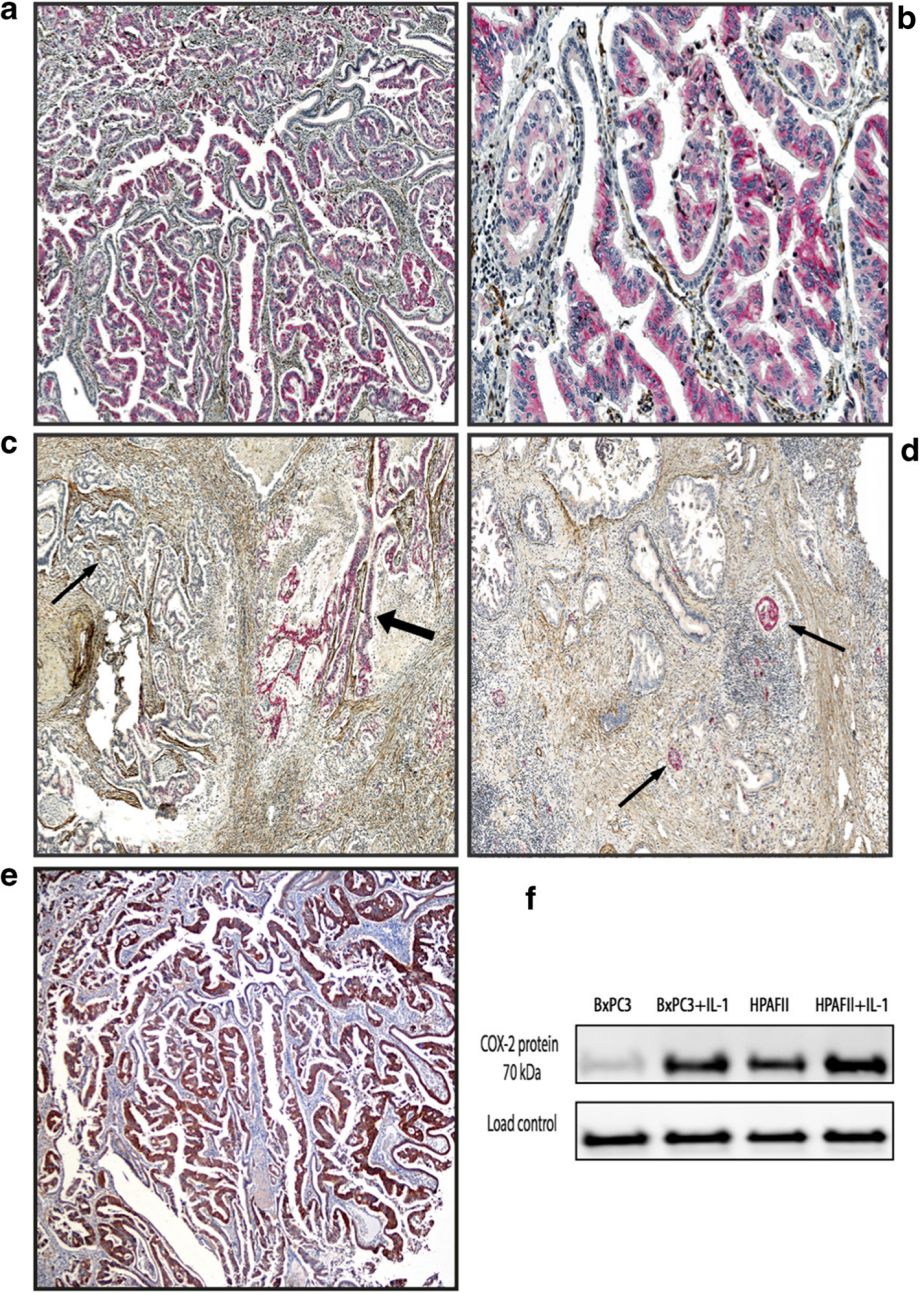
**COX-2 expression in tumour tissue from pancreatic cancer. a**-**d** Double immunostaing with monoclonal anti-COX-2 antibody (Thermo Fischer Scientific rabbit) and monoclonal anti-αSMA (Dako). COX-2 tumour positive cells (red colour), αSMA positive stromal cells (brown colour). **a** magnification × 100, **b** magnification × 200, **c** Heterogeneity in COX-2 expression within pancreatic cancer tissue. Areas with moderate to strong staining (thick arrow) coexist with COX-2 negative areas (thin arrow), (magnification x 100) **d** Moderately to strong COX-2 staining in islet cells (thin arrow), pancreatic cancer negative for COX-2 staining, (magnification x 100). **e** Immunohistochemistry of COX-2 expression in tumour tissue from pancreatic cancer. Immunostaining with monoclonal COX-2 mouse antibody Invitrogen (the same tumour as in **a**), magnification x 100. **f** Western blot of COX-2 expression in the moderately differentiated pancreatic cancer cell lines BxPC3 and HPAFII known to overexpress COX-2, with and without induction by interleukin 1 (Il-1), showed a specific bond for COX-2 (70 kDA) (monoclonal COX-2 mouse antibody Invitrogen).

Immunohistochemistry was performed on whole tumour slices, which were assessed without prior knowledge of the clinical and pathological parameters. In each section, five different representative high-power fields (100×) with tumour infiltration were selected and examined by light microscopy. The intensity of staining was estimated on a scale from 1-3 (1-negative, 2-moderate, 3-strong). Cells were considered positive only if COX-2 intensity was moderate or strong. The extent of the immunolabeling was assessed as the percentage of positively stained tumour cells and was expressed on the scale from 1-3 where 1 represented less than 10% cells stained, 2 represented 10-50% and 3 over 50%. Since COX-2 demonstrated considerable heterogeneity within individual cases, the final immunoscore was obtained as the average of the numeric scores for five high-power fields of each case considered positive in intensity scoring. Based on histograms of the staining for all tumours, the optimal cut-off value for discrimination between negative and positive staining was found to be 1.4. Islets of Langerhans and mucosa of the duodenum were moderately to strongly positive for COX-2, including those tumours with no COX-2 expression, and served as internal controls. Identical sections with omission of the primary antibody were used as negative controls. To test the validity of the Thermo antibody used for the study cohort, we performed additional immunostaining with a different monoclonal COX-2 mouse antibody, Invitrogen (Camarillo, CA, USA), on duplicates of twenty pancreatic cancer slides from the study cohort. The results were identical (Figure 1a and e). As Thermo antibody was not suitable for western blotting (producer recommendation), only the Invitrogen antibody was subjected to analysis by western blotting. The results showed a highly specific bond for COX-2 (Figure 1f).

Almost half of study specimens (44%) were evaluated independently by two examiners (EP and AS) and kappa interobserver was 0.73, indicating substantial agreement (95% CI 0.6-0.9).

### Statistical analysis

Associations between variables were examined using Chi-square test, Fisher’s exact test and Mann-Whitney test. Continuous variables were reported as median with corresponding range or interquartile range (IQR). Unadjusted survival analysis was performed using the Kaplan-Meier method, comparing curves using log-rank test. Multivariable Cox regression analysis was used for adjusted survival analysis. Possible interactions were evaluated by inclusion of an interaction term in the models. For all tests, a two-sided p < 0.05 was considered statistically significant. Statistical analyses were performed in SPSS 19 for Windows (SPSS Inc., Chicago, IL).

## Results

The study cohort consisted of 230 patients consecutively resected for adenocarcinomas originating from the ampulla (AC) (n = 62, 27%), distal bile duct (DBC) (n = 76, 33%), or pancreas (PC) (n = 92, 40%). Median age at time of resection was similar for the three groups (67 years, range 37-83; p = 0.463 Kruskal-Wallis). Overall 5-year (actual) survival was 5% for PC, 16% for DBC, and 44% for AC (p < 0.001).

### COX-2 expression and prognosis in ampullary, distal bile duct and pancreatic cancer

**Figure 2:**
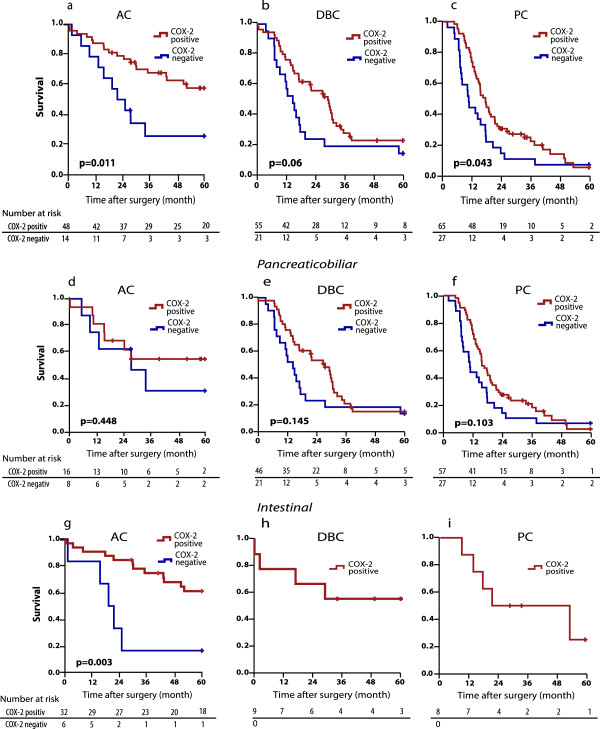
**Overall survival analysis stratified by COX-2 expression. a** Ampullary cancer (AC), **b** Distal bile duct cancer (DBC), **c** Pancreatic cancer (PC). **d**-**f** Overall survival analysis for AC, DBC and PC with pancreatobiliary differentiation stratified by COX-2 expression. **g**-**i** Overall survival analysis for AC, DBC and PC with intestinal differentiation stratified by COX-2 expression.

COX-2 staining was very similar in all three tumour types, with a positivity rate of 71% in PC, 72% in DBC, and 77% in AC. The COX-2 expression was detected in the cytoplasm of cancer cells in all three types of adenocarcinoma. No COX-2 immunostaining was detected in the stroma cells (Figure 1a,b, and e). The expression pattern showed heterogeneity both among different tumours and within the individual tumour, as areas with moderate to strong staining coexisted with negative areas within the same tumour (Figure 1c). Islet cells expressed moderately to strong COX-2 staining in all cases including those with no COX-2 expression in the tumour (Figure 1d). Irrespective of tumour origin, overall patient survival was more favourable in COX-2 positive than COX-2 negative tumours (Figure 2a-c). This was particularly prominent in AC (p = 0.011) and PC (p = 0.043) whereas the same tendency was seen in DBC although not reaching significance (p = 0.06). COX-2 expression varied according to the type of histological differentiation. In tumours with pancreatobiliary type of differentiation, two thirds of the tumours were COX-2 positive irrespective of anatomical origin (67%, 69%, and 68% in AC, DBC and PC, respectively). However there was no significant difference in overall survival when comparing COX-2 positive and negative tumours in this group (Figure 2d-f). All PC and DBC tumours with intestinal type of differentiation were COX-2 positive whereas 84% of the intestinal AC tumours expressed COX-2. The survival data of the intestinal AC tumours showed a favourable prognosis for patients with tumours expressing COX-2 (p = 0.003) (Figure 2g-i).

### Factors associated with prognosis in pancreatic adenocarcinoma

COX-2 expression status was compared across clinical parameters associated with survival in the subgroup consisting of the 92 patients resected for pancreatic adenocarcinoma. The median survival for patients with COX-2 positive tumours was 18 months (95% CI 14-22) as compared to 11 months (95% CI 9.6-12) for patients with COX-2 negative tumours (p = 0.043). COX-2 positive tumours were more likely associated with high degree of differentiation (p = 0.002) and with intestinal type of differentiation, although, the latter did not reach significance (p = 0.099) (Table 1) probably due to the low number of tumours of the intestinal differentiation type.

**Table 1:** Clinicopathological variables in 92 consecutive pancreatoduodenectomies for pancreatic cancer stratified by COX-2 status

**Characteristic**	**n(%)**	**COX2-neg. n(%)**	**COX2-pos. n(%)**	**p**^**a**^
COX-2				
Positive	65 (71%)			
Negative	27 (29%)			
Tumour size				
≤ 20 mm	15 (16%)	3 (20%)	12 (80%)	
> 20 mm	77 (84%)	24 (31%)	53 (69%)	0.54^b^
Lymph node metastasis				
N0, n (%)	25 (27%)	5 (20%)	20 (80%)	
N1, n (%)	67 (73%)	22 (33%)	45 (67%)	0.229
Lymph node ratio (LNR)^c^				
≤ 0.2	54 (59%)	13 (24%)	41 (76%)	
> 0.2	37 (41%)	13 (36%)	24 (65%)	0.251
Vascular invasion				
No, n (%)	30 (33%)	12 (40%)	18(60%)	
Yes, n (%)	62 (67%)	15 (24%)	47 (76%)	0.119
Perineural infiltration				
No, n (%)	15 (16%)	3 (20%)	12 (80%)	
Yes, n (%)	77 (84%)	24 (31%)	53 (69%)	0.54^b^
T classification				
T1	3 (3%)	1 (33%)	2 (67%)	
T2	6 (7%)	1 (17%)	5 (83%)	
T3	83 (90%)	25 (30%)	58 (70%)	0.851^b^
R1 resection status, n (%)				
R0, n (%)	40 (44%)	10 (25%)	30 (75%)	
R1, n (%)	52 (56%)	17 (33%)	35 (67%)	0.422
Degree of differentiation				
Grade I, II	53 (58%)	9 (17%)	44 (83%)	
Grade III, IV	39 (42%)	18 (46%)	21 (54%)	0.002
Type of differentiation				
Pancreaticobiliary, n (%)	84 (91%)	27 (32%)	57 (68%)	
Intestinal, n (%)	8 (9%)	0 (0%)	8 (100%)	0.099^b^

PC, pancreatic adenocarcinoma.

^a^Chi-square test, when not otherwise specified.

^b^Fisher’s Exact Test.

^c^LNR assessment of 91 patients since in one specimen no lymph nodes were retrieved.

**Figure 3:**
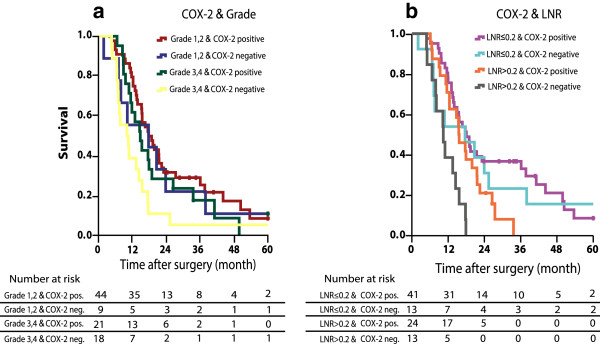
Overall survival analysis for patients with pancreatic cancer stratified by COX-2 expression and a degree of differentiation, b Lymph node ratio (LNR).

There was no association with COX-2 positivity and R-status, lymph node ratio (LNR), lymph node status, tumour diameter, T classification, and vascular or perineural infiltration (Table 1). Since tumours expressing COX-2 were significantly more likely to be highly differentiated than COX-2 negative tumours, the joint effects of COX-2 status and differentiation grade on survival were assessed by Kaplan-Meier analysis, stratifying for COX-2 status (positive vs. negative) and differentiation grade (grade 1 and 2 vs. grade 3 and 4) (Figure 3a). Patients whose tumours did not express COX-2 and had a low differentiation grade (grade 3 and 4) had significantly poorer survival than the other three groups (p = 0.006).

In a previous report we found that LNR independently predicted prognosis in a multivariate model for survival in resected pancreatic cancer [38]. We thus also examined the joint effects of COX-2 status and LNR, and found that patients with COX-2 negative tumours and LNR >0.2 had significantly worst prognosis (p < 0.001) (Figure 3b).

In a multivariate analysis model including COX-2 expression, LNR, tumour size, margin status, vascular and perineural infiltration, COX-2 negative tumours and LNR > 0.2 independently predicted poor prognosis (Table 2). Since there was a strong correlation between COX-2 expression and differentiation grade (p = 0.002) it was not possible to include differentiation grade in the same model.

**Table 2:** Multivariate Cox regression analysis of histopathologic factors in 92 patients with pancreatic cancer

	**p-value**	**HR**	**95% CI**
R-status (R1vs R0)	0.87	1.038	0.65 - 1.65
Vascular invasion (Involved vs non- involved)	0.455	1.208	0.74 - 1.98
Perineural infiltration (Involved vs non- involved)	0.359	1.369	0.70 - 2.68
Tumour size (> 20 mm vs ≤ 20 mm)	0.315	1.434	0.71 - 2.90
COX-2 expression (Negative vs Positive)	0.047	1.642	1.01 - 2.68
Lymph node ratio (LNR) (> 0.2 vs ≤ 0. 2)	0.032	1.757	1.05 - 2.94

Only a minority of the patients received adjuvant chemotherapy. Although the numbers are small, there was no difference in survival between patients with COX-2 positive and COX-2 negative tumours who received adjuvant treatment.

## Discussion

There is a large body of epidemiological, clinical and molecular evidence suggesting that COX-2 is implicated in the oncogenesis and progression of gastrointestinal malignancies, including adenocarcinomas derived from pancreatic head structures. It has previously been shown that COX-2 is upregulated in subsets of pancreatic, ampullary and distal bile duct adenocarcinomas although the proportion of upregulated tumours varies in the different reports. Furthermore, data on the prognostic importance of COX-2 expression in these tumours is conflicting. In pancreatic adenocarcinoma, two studies reported that COX-2 expressing tumours were associated with worse overall prognosis [19,21] whereas other studies have suggested a trend towards better prognosis for tumours with high COX-2 expression [22] or no association at all [39-41]. The present data on pancreatic, distal bile duct and ampullary adenocarcinomas indicates a more favourable overall survival for patients with COX-2 expressing tumours.

In periampullary and pancreatic head tumours, we have previously shown that histologic subtyping of these tumours into intestinal and pancreatobiliary types correlates with cell-type specific markers [36] and prognosis [3,37]. As COX-2 is thought to be expressed in epithelial cells throughout the gastrointestinal tract [5,12,42] it was of particular interest to examine whether there are differences in COX-2 expression in the intestinal and pancreatobiliary subtypes. Of note, most intestinal ampullary tumours (84%) were COX-2 positive, and in particular, all intestinal pancreatic and distal bile duct tumours were COX-2 positive. Patients with ampullary cancers of the intestinal subtype, which expressed COX-2, had a favourable prognosis with a 5-year actual survival of 60%. Histopathologic type of differentiation combined with biomarkers or gene expression profiles has recently attracted interest as important factors for outcome as well as stratification for adjuvant chemotherapy in ampullary adenocarcinoma [43,44].

The finding in the present study that COX-2 expression correlates with a favourable prognosis in pancreatic cancer can be explained by the fact that there is a statistically significant association between COX-2 positivity and high degree of differentiation. More than 80% of tumours with high differentiation grade showed overexpression of COX-2. This result is consistent with previous observations from studies of cultured pancreatic cancer cells and pancreatic cancer tissue. In cultured tumour cells COX-2 expression was found to be restricted to moderately and highly differentiated pancreatic cancer cell lines [23,26,45]. In human pancreatic adenocarcinoma tissue, well differentiated lesions expressed COX-2 to the highest degree, whereas there was less expression of COX-2 in moderately and poorly differentiated lesions [30]. In our study, the subgroup of patients with COX-2 positive/well differentiated tumours had a significantly better survival compared to patients with COX-2 negative/poorly differentiated tumours, whereas COX-2 positive/poor differentiation and COX-2 negative/high differentiation formed an intermediate group with respect to survival. Thus the presence of COX-2 expression in these tumours appears to be a marker of favourable prognosis closely linked to the degree of tumour differentiation. Consistent with the latter the strong statistical association between COX-2 expression and differentiation grade precluded inclusion of both variables in the same multivariable model for survival.

The precise function of COX-2 in pancreatic cancer development is not known. In the normal pancreas, only islet cells always express COX-2 [24]. In transgenic mice models, overexpression of COX-2 in normal pancreatic ductal cells results in development of dysplastic changes resembling IPMNs and PanINs [46,47] suggesting a primary role of pancreatic cell COX-2 overexpression in the initiation of ductal adenocarcinoma. Recent evidence suggests that this is an intrinsic role of pancreatic cells independent of prostaglandins from the tumour microenvironment [48]. These observations support the concept that COX-2 overexpression might be a causal factor in pancreatic cancer development. It has also been suggested that pancreatic cancers that lack COX-2 (and COX-1) depends on exogenic prostaglandins from stromal fibroblasts for proliferation and other cancer-promoting effects [49]. Since COX-2 overexpression is implicated in tumour development, its expression in pancreatic cancer was hypothesized to result in a poor patient prognosis [19]. This hypothesis is difficult to reconcile with the observation that in fully developed tumours, COX-2 expression has been shown to be a function of differentiation status, with highest expression in well differentiated tumours [30]. In addition, it has been demonstrated that COX-2 expression varies markedly throughout the pathological process of pancreatic neoplasia. COX-2 expression increases in a stepwise manner with each initial stage of neoplastic progression up to the PanIN 2 stage, whereas COX-2 expression was relatively lower in invasive cancers [30].

Some of the discrepancies in results between our study and the studies by Juuti et al [19] and Matsubayashi et al [21] might be explained by methodological differences in patient sampling and/or tumour immunohistochemistry techniques. Since it is well known that it can be difficult to determine the precise anatomical origin of tumours of the pancreatic head, all cancers in the present series were re-evaluated for correct sub-classification into ampullary, distal bile duct or pancreatic tumours. There are also certain differences pertaining to the immunohistochemistry protocols that differ in our study compared to the studies by Juuti et al [19] and Matsubayashi et al [21]. In the work of Juuti, more than 30 years old specimens were included in the study cohort. It is known that for immunohistochemical staining protocols aging of fixed tumour tissue might interfere with staining [50]. Not only aging of the waxed specimen itself, but also variations in fixation protocols over time may result in inadequate staining. This may partly explain the low frequency of COX-2 staining (36%) in their data, compared to 55-80% in most other reports [20,22,23,25,26,41,51]. Since COX-2 expression in pancreatic tumours often is heterogeneous [24,29,30], the actual number of COX-2 positive tumours might be underestimated unless immunohistochemistry is performed on whole slide sections and assessed on multiple different high-power fields within each tumour. In the study of Matsubayashi [21], assessment of COX-2 staining was performed on tissue microarrays. Although this method has many advantages, tissue microarrays might not be the optimal method for assessment of COX-2 staining even if two cores of tumour tissues were studied from each tumour. This may partly explain the lower proportion of tumours expressing COX-2 in some studies [19,21] and hence the differences in patient survival.

## Conclusion

COX-2 is overexpressed in pancreatic cancer, ampullary cancer and distal bile duct cancer and confers a survival benefit in all three cancer types. The overexpression is consistently linked to the histopathological type of differentiation and to the degree of differentiation. In pancreatic adenocarcinoma, COX-2 overexpression independently predicts a favourable prognosis.

## Competing interests

The authors declare that they have no competing interests.

## Authors’ contributions

EP, OPC, IPG conceived and planned the study. EP and IPG conducted acquisition of data. EP, ARS, and OPC performed immunohistochemistry. EP, ARS, OPC and IPG analysed and discussed the results. EP and IPG drafted the manuscript. All authors critically revised and approved of the final manuscript.

## Pre-publication history

The pre-publication history for this paper can be accessed here:

http://www.biomedcentral.com/1471-2407/14/458/prepub
